# Health-related quality of life among multiethnic public polyclinic users in Singapore and associated factors: a cross-sectional study

**DOI:** 10.1007/s11136-025-04069-9

**Published:** 2025-09-27

**Authors:** Ling Jie Cheng, Justin Guang Jie Lee, Calvin Wei Jie Chern, Jing Ying Cheng, Annushiah Vasan Thakumar, Nan Luo, Gerald Choon Huat Koh, Qin Xiang Ng

**Affiliations:** 1 Alice Lee Centre for Nursing Studies, Yong Loo Lin School of Medicine, National University of Singapore, Block MD6, Level 5, 14 Medical Drive, Singapore, 117599 Singapore; 2 National Perinatal Epidemiology Unit, Nuffield Department of Population Health, University of Oxford, Old Road Campus, Oxford, OX3 7LF UK; 3 Saw Swee Hock School of Public Health, National University of Singapore, Singapore, Singapore; 4 Khoo Teck Puat Hospital, Yishun Health, National Healthcare Group, Singapore, Singapore; 5 School of Pharmacy, Faculty of Health and Medical Sciences, Taylor’s University, Selangor, Malaysia; 6 Yong Loo Lin School of Medicine, National University of Singapore, Singapore, Singapore

**Keywords:** Health-related quality of life, Polyclinic users, Patient engagement, Patient activation

## Abstract

**Purpose:**

Health-related quality of life (HRQoL) is a crucial health outcome, reflecting both clinical status and broader well-being. Singapore faces the dual challenge of rapid population ageing and increasing chronic disease prevalence. Yet, comprehensive data on HRQoL in primary care remain scarce, as most research focuses on hospital-based or disease-specific populations rather than routine public primary care (polyclinic) users. This study aimed to describe HRQoL among public polyclinic users in Singapore and identify sociodemographic, clinical, and patient activation factors associated with HRQoL variations.

**Methods:**

We conducted a cross-sectional study among adults attending two large public polyclinics in Singapore. Participants completed the EQ-5D-5L, the Consumer Health Activation Index (CHAI), and surveys on sociodemographic and clinical characteristics. We summarised HRQoL and participant characteristics descriptively and used a two-part regression model for the EQ-5D-5L index and linear regression for the EQ VAS scores to identify independent predictors.

**Results:**

Among the 572 participants, the mean EQ index and EQ VAS scores were 0.89 (SD = 0.16) and 77.0 (SD = 12.7), respectively. Pain/Discomfort and Anxiety/Depression were the dimensions with the most reported problems (23.1% and 18.4%, respectively). Higher CHAI scores (EQ index: β = 0.002; VAS: β = 5.0) and better self-rated health (EQ index: β = 0.09; VAS: β = 14.3) predicted better HRQoL. Malay ethnicity and comorbidities were linked to lower EQ index scores, while younger age, male gender, and moderate income were associated with lower EQ VAS scores.

**Conclusions:**

HRQoL among Singaporean polyclinic users varies by sociodemographic, clinical, and activation factors, underscoring persistent age, ethnic, and gender disparities. Integrating HRQoL assessment and culturally tailored interventions into primary care may help reduce these inequities.

**Supplementary Information:**

The online version contains supplementary material available at 10.1007/s11136-025-04069-9.

## Introduction

Health-related quality of life (HRQoL) is increasingly recognised as a critical outcome in healthcare evaluation, particularly as health systems worldwide face the dual challenge of population ageing and the increasing burden of chronic disease. In Singapore, projections indicate that one in four citizens will be aged 65 years or older by 2030. These demographic shifts are placing unprecedented demands on primary care which serves as the cornerstone of chronic disease management and preventive health [1]. Despite the growing prominence of measuring HRQoL, there remains a notable lack of robust, population-based data on the quality of life among primary care users in Singapore. Most existing research has focused on hospital-based or disease-specific cohorts, such as those with diabetes or cardiovascular disease, limiting the generalisability of findings to the broader, more diverse primary care population [2].

While international studies have identified several common determinants of HRQoL in primary care, findings also vary across contexts and populations. Studies from Brazil, Spain, Hong Kong, Sweden, and India have consistently identified older age, female gender, higher multimorbidity and unemployment as significant predictors of poorer HRQoL [3–6]. Lower socioeconomic status and poor self-reported health are also frequently associated with worse HRQoL [3, 6]. However, the impact of higher education is less consistent. Some studies report an association between higher education and better HRQoL [3, 7], while others find no significant relationship [8, 9]. Despite these insights, a research gap remains in understanding how modifiable factors, particularly health activation, defined as an individual’s knowledge, skills and confidence in managing their health [10], interact with sociodemographic and clinical characteristics to shape HRQoL outcomes. Although previous research suggested that health activation can influence both HRQoL and healthcare utilisation [11, 12], its precise role and interaction with established determinants have not been fully understood.

Most HRQoL research in Singapore has revolved around the general population [9, 13].For example, Tan, Lim [9] established population norms with the EQ-5D-5L Index (EQ index), although they used an interim value set rather than the final version later published by Luo et al. [14], while Leow, Griva [13] used the SF-36 in a national cohort study. In contrast, research in primary care settings has been more limited and typically confined to specific subgroups, such as older adults with multimorbidity [15] and patients with chronic wounds [16]. Notably, Quah, Wang [15] applied the EQ-5D-3L crosswalk value set, and Zhu, Olsson [16] reported only domain-level responses and EQ VAS scores. This methodological variation, alongside the predominance of either general population samples or narrowly defined clinical groups, highlights a significant gap. There remains a need for robust, contextually relevant research using the finalised Singapore EQ-5D-5L value set [14] to comprehensively examine the determinants of HRQoL in the broader and more diverse primary care population. Therefore, this study aims to generate contextually relevant HRQoL estimates for public polyclinic users in Singapore by systematically examining the associations of sociodemographic, clinical, and health activation factors with HRQoL.

## Methods

This study was reported in accordance with the STROBE (Strengthening the reporting of observational studies in epidemiology) checklist (Supplementary Table S1) [17].

### Study design, setting, and participants

This cross-sectional study analysed secondary data from a larger validation project of the Consumer Health Activation Index (CHAI) conducted in Singapore [18]. Participants were recruited using quota sampling from two public primary care polyclinics located in the western region of Singapore between October 2024 and December 2024.

These polyclinics are part of the National University Polyclinics (NUP), a network managed under the National University Health System (NUHS), which is one of Singapore’s three integrated public healthcare clusters [19]. In Singapore, polyclinics are government-funded primary care centres that provide a comprehensive range of medical services, including acute care, chronic disease management, preventive health, and health education to the community. These polyclinics serve as a key access point for subsidised healthcare and are designed to cater to the needs of Singapore’s multiethnic urban population [20].

The two selected polyclinics were chosen for their demographic representativeness and their major role in delivering primary care to residents in the west of Singapore, a region that has seen rapid urban development and population growth. Participants were eligible for inclusion if they were aged 21 years or older and able to communicate effectively in English. Both the EQ-5D-5L and the CHAI were self-administered in paper form during the study visit.

### Measures

*EQ-5D-5L*: This validated instrument assessed HRQoL across five dimensions (mobility, self-care, usual activities, pain/discomfort, anxiety/depression) using a five-level Likert scale [21]. Each unique combination of responses defines a health state, and the EQ-5D-5L can describe 3,125 possible health states. For example, “11111” represents no problems in all five dimensions, while “55555” represents extreme problems in all five dimensions [22]. The index score (range: − 0.85–1.0) was calculated using the Singapore-specific value set [14]. A score of 1.0 represents full health, 0 corresponds to a health state equivalent to dead, and negative values indicate health states considered worse than dead. The EQ VAS (0–100) captured subjective health perceptions, with 0 representing the worst imaginable health and 100 the best imaginable health [22]. The EQ-5D-5L demonstrates robust psychometric properties in diverse Asian populations and primary care settings, with strong reliability and validity [23–25].

*CHAI*: The 10-item CHAI is a freely available, 10-item instrument designed to assess an individual’s knowledge, skills, and confidence in managing their health, reflecting their level of health activation [26]. Each item is rated on a six-point Likert scale, with total scores transformed to a 0–100 scale; higher scores indicate greater health activation. In Singapore, the CHAI has demonstrated a unidimensional structure, strong internal consistency (Cronbach’s α = 0.85), and good test–retest reliability (ICC = 0.80), supporting its validity for use in primary care populations [18].

*Covariates*: A structured questionnaire captured sociodemographics (age, gender, ethnicity, education, employment), clinician-diagnosed chronic conditions, and self-rated health (5-point Likert scale). The selection of these covariates was informed by existing literature, which consistently identifies sociodemographic factors (such as age, gender, ethnicity, employment, and education), multimorbidity, and self-rated health as determinants of HRQoL in primary care populations [3–9]. In addition, we included health activation as a covariate, given prior evidence of its influence on both HRQoL and healthcare utilisation [10–12].

### Sample size calculation

The minimum required sample size was estimated using G*Power version 3.1 [27], based on a linear multiple regression model with up to 15 predictors. Assuming a medium effect size (f^2^ = 0.15), an alpha level of 0.05 and 90% power, the required sample size was 390 participants. This effect size was chosen based on prior studies examining predictors of HRQoL in primary care settings [9, 15]. To account for potential item-level missingness and to support subgroup and sensitivity analyses, we aimed to recruit at least 500 participants. Ultimately, 572 participants were included in the analysis, providing adequate power to detect meaningful associations across all models.

### Statistical analysis

Descriptive statistics were used to characterise the sample and distributions of HRQoL measures. Univariate analyses, including independent t-tests and analysis of variance, were conducted to identify subgroup differences. Pearson correlation tests were performed to explore associations between continuous variables. Given the left-skewed distribution of the EQ index and a substantial ceiling effect, with 41.8% of respondents reporting full health, a two-part modelling strategy was applied in line with best practices for analysing utility data [28]. The first part employed logistic regression to estimate the likelihood of reporting full health (EQ index equal to one), while the second part used generalised linear regression with a gamma distribution and log-link function to analyse scores below one, thus accounting for the skewed and bounded nature of the data. The EQ VAS, which was approximately normally distributed according to the Shapiro–Wilk test (p = 0.12), was analysed using linear regression. For both EQ index and EQ VAS models, variables were selected using backward stepwise regression with a removal threshold of *p* > 0.10 to derive the final multivariable models. Analyses were performed in STATA 18.0 with statistical significance set at *p* < 0.05.

### Ethical considerations

The study received approval from the Department Ethics Review Committee, National University of Singapore on 04 June 2024 (approval number SSHSPH-269). Written informed consent emphasised voluntary participation and data anonymisation. Potential self-report bias was minimised using pilot-tested surveys and trained interviewers.

## Results

### Participants

A total of 572 adult participants were included in the analysis, reflecting the sociodemographic diversity of Singapore’s public polyclinic users. The mean age was 47.0 years (SD = 16.6), with a slight predominance of females (51.9%, n = 297). Ethnic representation was broadly consistent with national demographics: 69.0% Chinese, 17.0% Malay, 9.8% Indian, and 4.2% other ethnicities. To contextualise representativeness, we also compared our sample’s educational attainment with Singapore’s general population characteristics using national 2024 population estimates [29]. Chronic conditions were common in the study sample, with hyperlipidaemia (28.5%, n = 163), hypertension (27.5%, n = 157) and diabetes (18.0%, n = 103) being the most frequently reported diagnoses (Table 1).

**Table 1:** Socio-demographic and health-related characteristics of study participants (N = 572)

**Variables**	**Analytic sample (n (%)**	**Singapore 2024 population estimate (%)** ^**2**^
**Health Activation**^**1**^, **Mean (SD)**	77.9 (10.9)	
**Body mass index, Mean (SD)**	25.4 (5.2)	
**Age, Mean (SD)**	47.0 (16.6)	
21–44	263 (46.0)	43.7
45–64	209 (36.5)	33.3
≥ 65	100 (17.5)	22.9
**Gender**		
Female	297 (51.9)	51.3
Male	275 (48.1)	48.7
**Ethnicity**		
Chinese	395 (69.0)	74.0
Malay	97 (17.0)	13.5
Indian	56 (9.8)	9.0
Others	24 (4.2)	3.4
**Education**		
Secondary or lower	129 (22.6)	
Post-secondary	230 (40.2)	
Tertiary	213 (37.2)	
**Housing type**		
HDB 1–4 room	243 (42.5)	
HDB 5 room/executive flat	202 (35.3)	
Private	127 (22.2)	
**Income level (SGD$)**		
< $3000	254 (44.4)	
$3000-$4999	138 (24.1)	
≥ $5000	180 (31.5)	
**Presence of comorbidities**		
No	261 (45.6)	
Yes	311 (54.4)	
**Presence of diabetes mellitus**		
No	469 (82.0)	
Yes	103 (18.0)	
**Presence of hypertension**		
No	415 (72.5)	
Yes	157 (27.5)	
**Presence of hyperlipidaemia**		
No	409 (71.5)	
Yes	163 (28.5)	
**Presence of gout**		
No	557 (97.4)	
Yes	15 (2.6)	
**Presence of other chronic disease**		
No	513 (89.7)	
Yes	59 (10.3)	
**Presence of multimorbidity (≥ 3 chronic conditions)**		
No	518 (90.6)	
Yes	54 (9.4)	
**In general, how would you rate your health?**		
Poor	4 (0.7)	
Fair	134 (23.4)	
Good	292 (51.1)	
Very Good	123 (21.5)	
Excellent	19 (3.3)	
**Compared to one year ago, how would you rate your general health right now?**		
Much worse than one year ago	12 (2.1)	
Somewhat worse than one year ago	98 (17.1)	
About the same	327 (57.2)	
Somewhat better now than one year ago	96 (16.8)	
Much better now than one year ago	39 (6.8)	

HDB: Housing Development Board; SGD: Singapore dollar; 1SGD = 0.78 US dollars; ^1^ CHAI: Consumer Health Activation Index (measure health activation); ^2^ Singapore Department of Statistics. (2024, September). *Population Trends, 2024* (20th ed.). Singapore Department of Statistics. Retrieved 25 June 2025, from https://www.singstat.gov.sg/-/media/files/publications/population/population2024.ashx

**Fig. 1:**
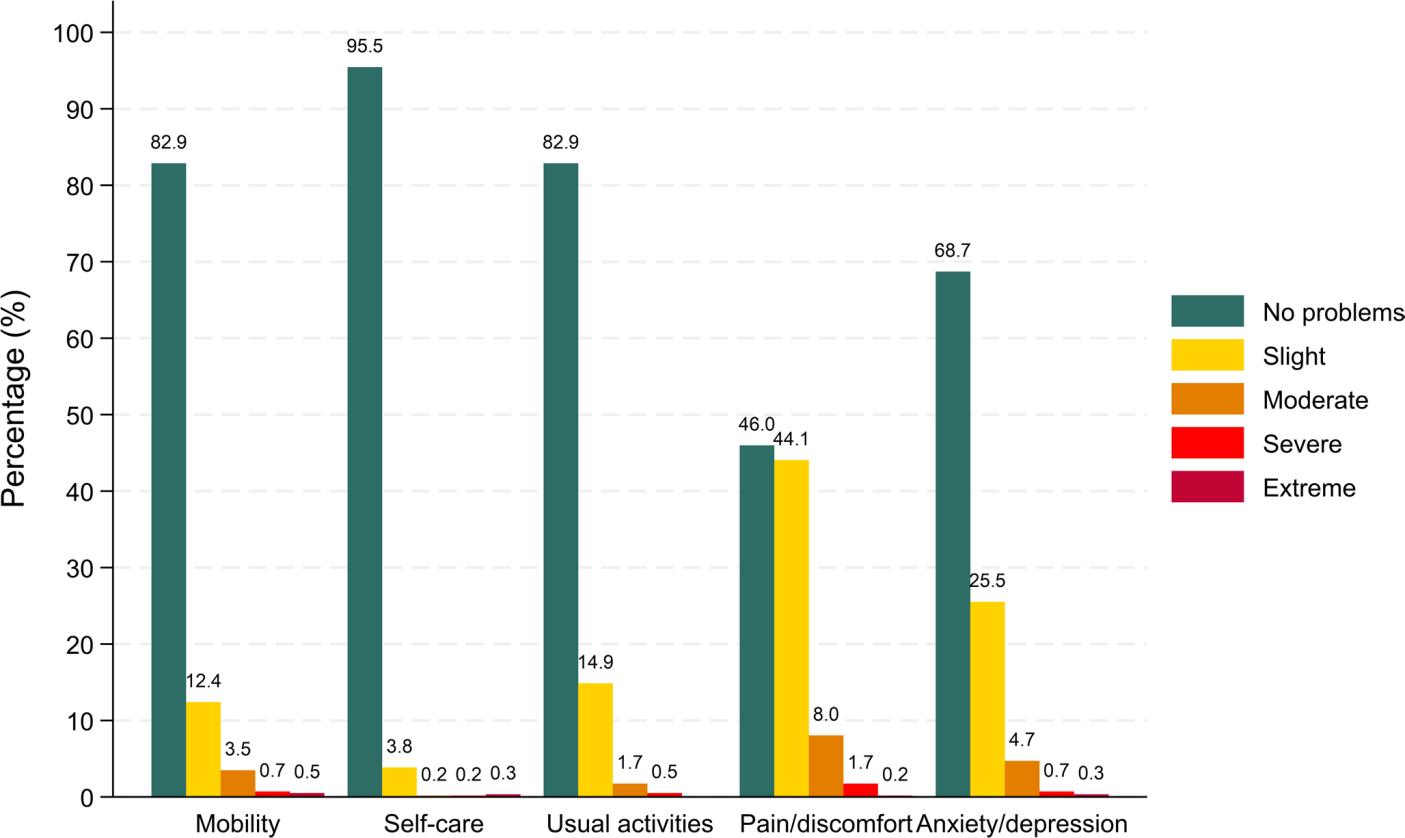
Percentage of polyclinic users in Singapore reporting each level of problems across the EQ-5D-5L dimensions

The overall mean EQ index score was 0.89 (SD = 0.16), and the mean EQ VAS score was 77.0 (SD = 12.7) (Table 2). Notably, 41.8% (n = 239) of respondents reported full health (i.e., health state “11111”). Figure 1 presents the distribution of response levels across the five EQ-5D-5L dimensions. “No problems” was the most frequently reported level across all domains, particularly in self-care (95.5%), mobility (82.9%), and usual activities (82.9%). However, 54.0% of respondents (n = 309) reported at least some pain/discomfort, and 31.3% (n = 179) reported at least some anxiety/depression.

**Table 2:** Comparison of mean scores of EQ index and EQ VAS among different sociodemographic and clinical characteristics subgroups

**Variables**	**EQ index**		**EQ VAS**	
	**Mean (SD)**	***P***-**value**	**Mean (SD)**	***P***-**value**
**Overall**	0.89 (0.16)		77.0 (12.7)	
**Age**				
21–44	0.91 (0.17)	< 0.001***	77.1 (12.6)	0.97
45–64	0.90 (0.12)		76.8 (12.8)	
≥ 65	0.84 (0.22)		77.0 (13.0)	
**Gender**				
Female	0.90 (0.15)	0.30	78.1 (12.5)	0.03*
Male	0.89 (0.18)		75.8 (12.9)	
**Ethnicity**				
Chinese	0.90 (0.15)	0.09	76.9 (12.1)	0.80
Malay	0.88 (0.14)		76.6 (13.6)	
Indian and others	0.86 (0.25)		77.8 (14.7)	
**Education**				
Primary or lower	0.83 (0.25)	< 0.001***	75.9 (13.6)	0.07
Secondary and post-secondary*	0.91 (0.14)		78.5 (12.4)	
Tertiary	0.92 (0.11)		76.0 (12.5)	
**Housing type**				
HDB 1–4 room	0.87 (0.18)	0.05	76.2 (13.0)	0.35
HDB 5 room/executive flat	0.90 (0.14)		77.1 (12.9)	
Private	0.91 (0.18)		78.3 (12.0)	
**Income level (SGD$)**				
< $3000	0.87 (0.21)	0.005**	76.6 (13.4)	0.15
$3000−$4999	0.91 (0.13)		78.8 (12.2)	
≥ $5000	0.92 (0.11)		76.1 (12.0)	
**Presence of comorbidities**				
No	0.92 (0.12)	< 0.001***	78.4 (12.8)	0.02*
Yes	0.87 (0.19)		75.8 (12.6)	
**Presence of diabetes mellitus**				
No	0.90 (0.15)	0.002**	77.5 (12.5)	0.02*
Yes	0.85 (0.21)		74.4 (13.4)	
**Presence of hypertension**				
No	0.90 (0.14)	0.005**	77.3 (12.7)	0.39
Yes	0.86 (0.21)		76.2 (12.9)	
**Presence of hyperlipidaemia**				
No	0.90 (0.16)	0.008**	77.5 (13.0)	0.15
Yes	0.86 (0.17)		75.8 (12.1)	
**Presence of gout**				
No	0.89 (0.16)	0.08	77.1 (12.7)	0.24
Yes	0.82 (0.22)		73.1 (12.7)	
**Presence of other chronic disease**				
No	0.90 (0.17)	0.03*	77.2 (12.8)	0.17
Yes	0.85 (0.15)		74.8 (11.6)	
**Presence of multimorbidity (≥ 3 chronic conditions)**				
No	0.90 (0.16)	< 0.001***	77.3 (12.6)	0.06
Yes	0.82 (0.22)		73.9 (13.3)	
**In general, how would you rate your health?**				
Poor/ Fair	0.83 (0.18)	< 0.001***	68.6 (12.4)	< 0.001***
Good	0.90 (0.17)		77.4 (11.8)	
Very Good/ Excellent	0.95 (0.10)		84.3 (9.8)	
**Compared to one year ago, how would you rate your general health right now?**				
Much/Somewhat worse than one year ago	0.81 (0.20)	< 0.001***	70.2 (12.4)	< 0.001***
About the same	0.91 (0.13)		78.4 (12.1)	
Much/Somewhat better now than one year ago	0.92 (0.18)		79.1 (12.8)	

*: < 0.05; **: < 0.01; ***: < 0.001; SD: Standard deviation; SGD: Singapore dollar; 1SGD = 0.78 US dollars

Significant disparities in HRQoL were observed across sociodemographic groups. Older adults aged 65 years and above reported lower mean EQ index scores (0.84, SD = 0.22) compared to those aged 21–44 years (0.91, SD = 0.17; p < 0.001), although EQ VAS scores were similar across age groups. Educational attainment showed a strong positive association with HRQoL: participants with tertiary education had the highest mean EQ index (0.92, SD = 0.11), while those with primary or lower education had the lowest (0.83, SD = 0.25; p < 0.001). Higher income was also linked to better outcomes, with those earning ≥ $5,000 SGD reporting a mean EQ index of 0.92 (SD = 0.11) compared to 0.87 (SD = 0.21) for those earning less than $3,000 (p = 0.005). Gender differences were evident in EQ VAS scores, with females reporting higher values (78.1, SD = 12.5) than males (75.8, SD = 12.9; p = 0.03). While Malay participants had slightly lower EQ index scores (0.88, SD = 0.14) than Chinese participants (0.90, SD = 0.15), this difference was not statistically significant (p = 0.09).

Clinical characteristics also played a substantial role in HRQoL outcomes. The presence of comorbidities was associated with lower EQ index (0.87, SD = 0.19 vs. 0.92, SD = 0.12; p < 0.001) and EQ VAS scores (75.8, SD = 12.6 vs. 78.4, SD = 12.8; p = 0.02). Participants with diabetes mellitus had significantly lower EQ index (0.85, SD = 0.20; p = 0.002) and EQ VAS (74.4, SD = 13.4; p = 0.02) compared to those without diabetes. Multimorbidity (≥ 3 chronic conditions) was associated with the lowest HRQoL, with mean EQ index and EQ VAS scores of 0.82 (SD = 0.22) and 73.9 (SD = 13.3), respectively (p < 0.001 for EQ index). Self-rated health was a strong determinant: those reporting poor or fair health had markedly lower EQ index (0.83, SD = 0.18) and EQ VAS (68.6, SD = 12.4) compared to those reporting very good or excellent health (EQ index: 0.95, SD = 0.10; EQ VAS: 84.3, SD = 9.8; both p < 0.001).

Correlation analyses (Table 3) revealed moderate positive associations between the EQ index and EQ VAS scores (r = 0.39). Health activation scores showed significant positive correlations with both EQ index (r = 0.17) and EQ VAS (r = 0.34) scores. Age was negatively correlated with EQ index (r =  − 0.15) but not with EQ VAS.

**Table 3:** Correlation matrix of EQ index, EQ VAS and other explanatory variables

**Variables**	**EQ index**	**EQ VAS**	**CHAI**	**BMI**	**Age**
EQ index	–				
EQ VAS	0.39***	–			
CHAI	0.17***	0.34***	–		
BMI	− 0.06	− 0.07	0.04	–	
Age	− 0.15***	− 0.01	0.13**	0.06	–

^**^: < 0.01; ***: < 0.001

### Multivariable analyses

The two-part multivariable regression model (Supplementary Table S2) identified several independent predictors of HRQoL, with marginal effects representing the combined influence on both the probability of perfect health and health utility magnitude among those with imperfect health. For the EQ index, the presence of comorbidities was associated with lower expected scores (marginal effect = − 0.03, 95% CI: − 0.05 to − 0.005, p = 0.03). Higher health activation levels were positively associated with better index scores (marginal effect = 0.002, 95% CI: 0.0004 to 0.004, p = 0.02). Malay ethnicity was associated with lower expected EQ index scores compared to Chinese participants (marginal effect = − 0.03, 95% CI: − 0.06 to − 0.004, p = 0.03). Participants reporting 'Very Good/Excellent' health status had substantially higher expected EQ index scores (marginal effect = 0.09, 95% CI: 0.06 to 0.12, p < 0.001) compared to those reporting 'Poor/Fair' health (Table 4).

**Table 4:** Factors associated with health-related quality of life measured by EQ index and EQ VAS

**Variables**	**EQ index**		**EQ VAS**	
	**Adjusted Β (95% CI)**	***P*** **-value**	**Adjusted Β (95% CI)**	***P*** **-value**
**Health activation**	0.002 (0.0004, 0.004)	0.02*	5.0 (3.2, 6.8)	< 0.001***
**Age**			0.07 (0.01, 0.1)	0.02*
**Presence of comorbidities**	− 0.03 (− 0.05, − 0.005)	0.03*		
**Gender** (Ref: Female)				
Male			− 2.0 (− 3.8, 0.2)	0.03
**Ethnicity** (Ref: Chinese)				
Malay	− 0.03 (− 0.06, − 0.004)	0.03*		
Indian/ Others	− 0.06 (− 0.12, 0.007)	0.08		
**Income level (SGD$) **(Ref: < $3000)				
$3000-$4999			2.6 (0.3, 5.0)	0.03*
≥ $5000			0.4 (− 1.7, 2.6)	0.70
**In general, how would you rate your health?** (Ref: Poor/ Fair)				
Good	0.05 (0.01, 0.09)	0.01*	7.0 (4.6, 9.3)	< 0.001***
Very Good/ Excellent	0.09 (0.06, 0.12)	< 0.001***	14.3 (11.4, 17.2)	< 0.001***

^*^: < 0.05; ***: < 0.001; CI: Confidence interval; Ref: Reference; SGD: Singapore dollar; 1SGD = 0.78 US dollars

In the EQ VAS model, older age (β = 0.07, 95% CI: 0.01 to 0.1, p = 0.021) and higher health activation (β = 5.0, 95% CI: 3.2 to 6.8, p < 0.001) were both significantly associated with higher self-rated health. Male gender was associated with slightly lower EQ VAS scores (β = − 2.0, 95% CI: − 3.8–0.2, p = 0.03). An income of SGD $3000–$4999 was associated with higher EQ VAS scores (β = 2.6, 95% CI: 0.3 to 5.0, p = 0.028), whereas income ≥ SGD $5000 was not significantly associated with EQ VAS scores. Self-rated health remained the strongest predictor, with participants reporting ‘Very Good/Excellent’ health showing substantially higher EQ VAS scores (β = 14.3, 95% CI: 11.4 to 17.2, p < 0.001).

## Discussion

This study provides new insights into the determinants of HRQoL among users of public polyclinic in Singapore, revealing important patterns that extend our understanding of HRQoL in high-income Asian healthcare contexts. The findings demonstrate that health activation, self-rated health, and ethnic background are key independent predictors of both preference-based and subjective measures of HRQoL, with implications for clinical practice and health policy.

The substantial ceiling effect observed in our EQ index, with 41.8% of respondents reporting perfect health, highlights a recognised limitation of preference-based measures in relatively healthy populations [30]. Although our two-part modelling approach addressed the statistical challenges posed by this distribution, the ceiling effect may obscure important variations in HRQoL among those classified as having "perfect" health. Research indicates that the EQ-5D-5L does not capture key quality of life domains such as fatigue, social functioning, and specific disease-related concerns, which can affect more than half of the patients even in primary care settings where subclinical symptoms or early-stage conditions are prevalent [31]. Furthermore, the stronger predictive power of self-rated health compared to clinical measures in our study further supports the need to capture broader psychosocial and functional dimensions. Taken together, these considerations suggest that while EQ-5D-5L remains valuable for comparability and economic evaluation, it may not fully reflect outcomes most relevant to primary care. Future studies could improve sensitivity by extending EQ-5D-5L with targeted bolt-ons or by combining it with broader generic (e.g., WHOQOL) or disease-specific instruments.

When contextualised against recently published Singapore population norms [9], our participants reported consistently lower EQ index and EQ VAS scores across age, sex, and ethnic groups. For example, the normative mean EQ index for adults aged 21–44 years was 0.98 compared with 0.91 in our sample, while among adults aged ≥ 65 years the normative mean was 0.94 versus 0.84. With respect to sex, normative data indicate that men and women have broadly similar EQ index values, but men report lower EQ VAS scores than women [9]; our findings are consistent with this pattern, with male participants reporting lower VAS scores than females. Similarly, Malay participants reported lower HRQoL than Chinese participants, in line with both our regression results and population norms [9]. These comparisons confirm that polyclinic users represent a less healthy segment of the community, reflecting their higher burden of multimorbidity and chronic disease.

Our finding that higher health activation correlates with better HRQoL aligns with global evidence on patient engagement [32]. This relationship may stem from improved self-efficacy in health behaviours (e.g., medication adherence, preventive care) and more collaborative patient-clinician communication, which fosters psychological ownership of health decisions [32, 33]. While previous studies have highlighted cultural barriers to activation in hierarchical healthcare systems, our findings uniquely demonstrate this association within Singapore’s multicultural context, where traditional deference to medical authority may otherwise limit patient participation [34]. These findings suggest that activation-enhancing interventions, including shared decision-making tools and health coaching programmes, could be especially valuable in Asian healthcare settings. The moderate correlation between CHAI scores and HRQoL measures (r = 0.17–0.34) further supports the link between health literacy, health activation and subsequent improvements in quality of life, indicating that interventions targeting health literacy may also yield downstream benefits for HRQoL.

Our observation that self-rated health emerged as the strongest predictor of both EQ index and EQ VAS scores is consistent with extensive literature demonstrating its robust association with morbidity and mortality outcomes [3, 35]. This finding is particularly noteworthy given that self-rated health and quality of life are conceptually distinct constructs, with quality of life being more strongly influenced by mental health factors [36] while self-rated health is more closely tied to physical functioning [37]. In Singapore’s achievement-oriented society, self-rated health perceptions may reflect broader psychosocial stressors related to work, family responsibilities and social expectations that are not captured by traditional clinical measures [38]. These findings suggest that routine assessment of self-rated health in primary care could serve as an efficient screening tool for identifying patients at risk of poor HRQoL outcomes.

The persistent association between Malay ethnicity and lower EQ-5D index scores, even after adjusting for sociodemographic and clinical factors, mirrors patterns observed in other multiethnic societies and is consistent with previous studies in Singapore [9, 13, 15, 16]. The persistence of ethnic disparities after controlling for comorbidities and socioeconomic factors suggests the influence of unmeasured structural determinants, such as barriers to healthcare access, cultural factors affecting care-seeking behaviour and potential systemic biases in healthcare delivery. One possible explanation for the observed disparities is the broader context of social determinants of health, where Malays in Singapore (like many ethnic minority groups globally) may face cumulative disadvantages that impact health equity [39]. Educational attainment in our sample differed significantly by ethnicity: only 21.7% of Malays had a university degree, compared to 40.3% of Chinese and 41.3% of Indian/other participants. Compared with the Singapore 2024 population estimate, which report tertiary education rates of 30.6% among Malays and 50.9% among Chinese [29], our findings suggest that Malay polyclinic users in particular may be less representative of the general Malay population. Epidemiological data also indicate a higher prevalence of chronic conditions including diabetes, hypertension, and cardiovascular disease among the Malay populations, as well as higher rates of smoking and obesity compared to other ethnic groups [40–43]. Further analysis of our dataset supports these findings, revealing that Malay participants have a higher prevalence of multimorbidity and comorbid conditions compared to other ethnic groups. International literature indicates that such intersecting clinical and social factors often translate into reduced HRQoL among minority groups. These findings emphasise the need for culturally responsive care and policies that address upstream determinants of health.

While the broader literature [6, 9, 13, 15] typically finds that men report higher self-rated health and HRQoL than women, our study observed that male gender was associated with lower EQ VAS scores. This contrasts with international studies from Brazil, Spain, Hong Kong, and Sweden [3, 4, 6, 7], which consistently reported female gender as a predictor of poorer HRQoL. Several factors may account for this unexpected result. In Singapore’s social milieu, men may experience heightened psychosocial stress related to work pressures, financial responsibilities, and societal expectations, which are not fully captured by traditional HRQoL instruments and may negatively influence their subjective health ratings [44]. Additionally, men may be less likely to seek help for mental health concerns due to stigma, potentially leading to under-recognition and poor management of issues that impact their perceived health status [45]. These findings suggest the need for more nuanced, culturally sensitive approaches to HRQoL assessment and highlight the importance of targeted interventions addressing psychosocial well-being and mental health support for men in primary care settings.

Similarly, although many local and international studies report older age as a predictor of poorer HRQoL [4, 6, 7, 9, 15, 16], our regression models revealed the opposite pattern, with older adults showing higher EQ VAS scores despite lower EQ index values. This paradox persists despite older adults (≥ 65 years) demonstrating substantially lower EQ-5D index scores (0.84 vs. 0.91 for 21–44), suggesting a decoupling of functional health status from subjective evaluations. In Singapore’s context, this paradox may reflect cultural narratives of resilience among older adults, where self-rated health perceptions incorporate psychosocial factors like familial support or life satisfaction more strongly than physical limitations [39]. Alternatively, older adults in this polyclinic-based sample may represent a "healthy survivor" cohort with adaptive coping strategies, a phenomenon observed in populations with high healthcare engagement [46]. This finding aligns with earlier work on health activation [10, 32], suggesting that interventions leveraging intrinsic resilience factors could mitigate HRQoL declines in ageing populations, even when objective health status deteriorates.

The cross-sectional design of this study limits causal inference, and the reliance on self-reported measures may introduce recall bias. Nevertheless, the use of validated instruments with established psychometric properties in Asian populations, including Singapore-specific value sets for the EQ-5D-5L, strengthens the validity of our findings. Another limitation is that only English-speaking participants were included. While this reflects the language requirement of the original validation study, it may have excluded perspectives from segments of the population who are less proficient in English, particularly older adults and certain ethnic minority groups. This could limit the generalisability of our findings to Singapore’s broader, multilingual population. Future research should employ longitudinal designs to examine how changes in health activation and other modifiable factors influence HRQoL trajectories over time. In addition, qualitative studies exploring the cultural and structural factors underlying observed age, gender, and ethnic disparities in HRQoL could inform the development of more targeted and effective interventions.

In conclusion, our study provides new insights into the complex interplay between health activation, sociodemographic factors and HRQoL in Singapore’s multicultural primary care context. By highlighting persistent age, ethnic and gender disparities, with certain findings contrary to international trends, and demonstrating the unique predictive value of self-rated health, our findings underscore the limitations of relying solely on conventional HRQoL instruments in diverse populations. These results emphasise the need for culturally responsive assessment tools and targeted interventions that address both structural and psychosocial determinants of health. They also support the integration of patient-reported outcomes into Singapore’s national primary care transformation efforts. Future research should build on these findings through longitudinal and qualitative approaches to better understand the underlying mechanisms and to inform more equitable, patient-centred care strategies in Asian healthcare settings.

## Supplementary Information

Below is the link to the electronic supplementary material.

Supplementary Material 1

## Data Availability

Data and materials are available to strengthen the transparency and reliability of the study. Additional data are available upon request by sending an email to the corresponding author.
