# Correction: Health-related quality of life among multiethnic public polyclinic users in Singapore and associated factors: a cross-sectional study

**DOI:** 10.1007/s11136-025-04094-8

**Published:** 2025-11-05

**Authors:** Ling Jie Cheng, Justin Guang Jie Lee, Calvin Wei Jie Chern, Jing Ying Cheng, Annushiah Vasan Thakumar, Nan Luo, Gerald Choon Huat Koh, Qin Xiang Ng

**Affiliations:** 1 Alice Lee Centre for Nursing Studies, Yong Loo Lin School of Medicine, National University of Singapore, Block MD6, Level 5, 14 Medical Drive, Singapore, 117599 Singapore; 2 National Perinatal Epidemiology Unit, Nuffield Department of Population Health, University of Oxford, Old Road Campus, Oxford, OX3 7LF UK; 3 Saw Swee Hock School of Public Health, National University of Singapore, Singapore, Singapore; 4 Khoo Teck Puat Hospital, Yishun Health, National Healthcare Group, Singapore, Singapore; 5 School of Pharmacy, Faculty of Health and Medical Sciences, Taylor’s University, Selangor, Malaysia; 6 Yong Loo Lin School of Medicine, National University of Singapore, Singapore, Singapore

**Correction to: Quality of Life Research**

10.1007/s11136-025-04069-9

In this article, reference 14 was mistakenly processed as reference 47 and also cited incorrectly as reference 47 in the text. The correct version of reference 14 is provided below.

Luo, N., Vasan Thakumar, A., Cheng, L. J., Yang, Z., Rand, K., Cheung, Y. B., & Thumboo, J. (2025). Developing an EQ-5D-5L Value Set for Singapore. PharmacoEconomics, 10.1007/s40273-025-01519-7. Advance online publication. 10.1007/s40273-025-01519-7

The original article has been corrected.

